# A network pharmacology approach to determine the underlying mechanisms of action of Yishen Tongluo formula for the treatment of oligoasthenozoospermia

**DOI:** 10.1371/journal.pone.0252906

**Published:** 2021-06-21

**Authors:** Yangdi Chen, Fanggang Bi, Zixue Sun

**Affiliations:** 1 Henan University of Chinese Medicine, Zhengzhou, Henan, P. R. China; 2 Department of Orthopedic Surgery, The First Affiliated Hospital of Zhengzhou University, Zhengzhou, P. R. China; 3 Department of Reproductive Medicine, Henan Province Hospital of Traditional Chinese Medicine (The Second Affiliated Hospital of Henan University of Chinese Medicine), Zhengzhou, Henan, P. R. China; King Abdulaziz University, Faculty of Pharmacy, SAUDI ARABIA

## Abstract

Oligoasthenozoospermia is a complex disease caused by a variety of factors, and its incidence is increasing yearly worldwide. Yishen Tongluo formula (YSTLF), created by Professor Sun Zixue, has been used to treat oligoasthenozoospermia in clinical practice for several decades with a good therapeutic effect. However, the chemical and pharmacological profiles of YSTLF remain unclear and need to be elucidated. In this study, a network pharmacology approach was applied to explore the potential mechanisms of YSTLF in oligoasthenozoospermia treatment. All of the compounds in YSTLF were retrieved from the corresponding databases, and the bioactive ingredients were screened according to their oral bioavailability (OB) and drug-likeness (DL). The potential proteins of YSTLF were obtained from the traditional Chinese medicine systems pharmacology (TCMSP) database and the Bioinformatics Analysis Tool for Molecular Mechanism of Traditional Chinese Medicine (BATMAN-TCM) database, while the potential genes of oligoasthenozoospermia were obtained from the GeneCards database and the DisGeNET database. The STRING database was used to construct an interaction network according to the common targets identified by the online tool Venny for YSTLF and oligoasthenozoospermia. The topological characteristics of nodes were visualized and analyzed through Cytoscape. Biological functions and significant pathways were determined and analyzed using the Gene Ontology (GO) knowledgebase, the Kyoto Encyclopedia of Genes and Genomes (KEGG) and Metascape. Finally, the disease-formula-compound-target-pathway network was constructed by Cytoscape. A total of 106 bioactive ingredients and 134 potential targets from YSTLF were associated with oligoasthenozoospermia or considered to be therapeutically relevant. Pathway analysis indicated that the PI3K/Akt, MAPK and apoptosis signaling pathways were significant pathways involved in oligoasthenozoospermia. In conclusion, the current study expounded the pharmacological actions and molecular mechanisms of YSTLF in treating oligoasthenozoospermia from a holistic viewpoint. The potential molecular mechanisms were closely related to antioxidative stress, antiapoptosis and anti-inflammation, with TNF, CCND1, ESR1, NFKBIA, NR3C1, MAPK8, and IL6 being possible targets. This network pharmacology prediction may offer a helpful tool to illustrate the molecular mechanisms of the Chinese herbal compound YSTLF in oligoasthenozoospermia treatment.

## Background

In recent years, the incidence of male infertility has increased each year due to the influences of environmental pollution, psychological hazards, drug abuse, unhealthy living habits and other factors. According to the World Health Organization (WHO), infertility currently affects nearly 15% of couples of childbearing age worldwide, with male factors accounting for more than 50% of these cases [1, 2]. Infertility, which seriously threatens human reproductive health, has become the third most difficult disease in the world, following only cardiovascular disease and cancer [3]. Among the many causes of male infertility, reduced sperm count and decreased sperm motility are two of the most common. The WHO has stated that asthenospermia is defined as having a rapid forward motile sperm (PR) of less than 32% or a total of forward motile sperm and nonforward motile sperm (PR+NP) of less than 40% within one hour after ejaculation with a semen density greater than 15×10^6^/ml. Moreover, semen density less than 15×10^6^/ml is considered oligozoospermia [4]. At present, there is no effective drug for the treatment of oligoasthenozoospermia (oligozoospermia combined with asthenospermia) in modern medicine. In recent years, a boom in assisted reproductive technology (ART) has solved certain fertility problems; however, there are still some defects, such as adverse reactions, genetic risk, high cost, and a low success rate [5]. Traditional Chinese medicine (TCM), under the guidance of holistic concepts and treatment based on syndrome differentiation, offers satisfactory therapeutic methods for the treatment of oligoasthenozoospermia with low adverse effects.

Yishen Tongluo formula (YSTLF), an empirical formula, was created by Professor Sun Zixue, a director of reproductive medicine department of Henan Provincial Hospital of Traditional Chinese Medicine. YSTLF is composed of seven herbs, including Semen Cuscutae (Tu Si Zi; TSZ), Herba Epimedii (Yin Yang Hou; YYH), Radix Rehmanniae Preparata (Shu Di Huang; SDH), Radix Astragali (Huang Qi; HQ), Salvia Miltiorrhiza (Dan Shen; DS), Leech (Shui Zhi; SZ), and Radix Cyathulae (Chuan Niu Xi; CNX). Previous clinical trials have demonstrated that YSTLF can significantly improve semen density, a/a+b or PR/PR+NP sperm, and sperm motility (total effective rate: 89.74% vs 64.10% (p<0.05)) [6]. Despite the above findings, the molecular mechanisms underlying the therapeutic effects of YSTLF on oligoasthenozoospermia remain unclear. Thus, further research with the appropriate approaches is warranted to comprehensively reveal the involved potential mechanisms.

With a surge of progress in bioinformatics, network pharmacology has opened up a new field of pharmacological research. Based on the theories of multidirectional pharmacology and systems biology, network pharmacology can construct complex network models to study the biological or pharmacological properties of a target and explore its physiological or pharmacological mechanism supported by high-throughput data analysis, virtual computing technology and network public databases, etc [7, 8]. The systematic and holistic characteristics of network pharmacology correspond with the core ideas of the holistic philosophy of traditional Chinese medicine (TCM). Network pharmacology can be applied to illustrate the interactive relationship between multiple components, targets and pathways of bioactive compounds in TCM herbal medicines [9], which could help to evaluate the compatibility and rationality of Chinese medicinal formulae. Therefore, an increasing number of studies have depended on network pharmacology to probe the potential targets and molecular mechanisms of TCM herbal medicines on holistically complex diseases. Zhang et al. [10] relied on network pharmacology analysis and reported that Bushen Tiansui formula might treat Alzheimer’s disease mainly through the TNF and PI3K/Akt signaling pathways. Lu et al. [11] demonstrated that Xijiao Dihuang Decoction (25 g/kg) could improve survival after sepsis by regulating the NF-κB and HIF-1α signaling pathways based on network pharmacology analysis.

**Fig 1:**
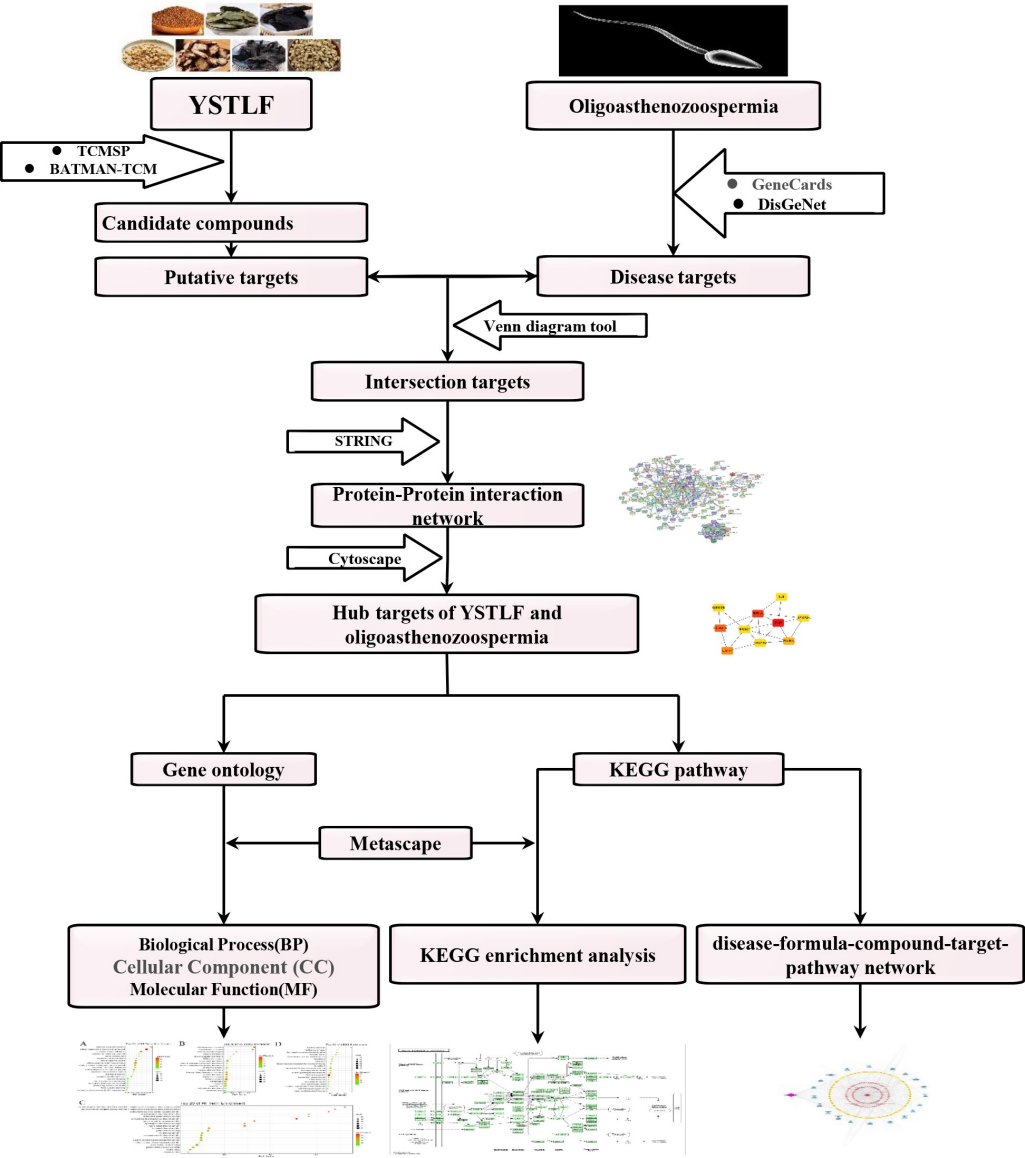
Workflow for elucidation of YSTLF in the treatment of oligoasthenozoospermia.

Therefore, in this study, public database resources and computational tools were used to investigate the pharmacological network of YSTLF in oligoasthenozoospermia by a network pharmacology approach. The research purpose was to elucidate the potential mechanism of YSTLF in the treatment of oligoasthenozoospermia through a prediction of the bioactive ingredients and a bioinformatics analysis of common targets. The detailed network pharmacology strategy of the current study is presented in **Fig 1**.

## Materials and methods

### YSTLF bioactive ingredients

All of the compounds of each herb in YSTLF were retrieved from the traditional Chinese medicine systems pharmacology (TCMSP) database (http://lsp.nwu.edu.cn/tcmsp.php) and the Bioinformatics Analysis Tool for Molecular Mechanism of Traditional Chinese Medicine (BATMAN-TCM) database (http://bionet.ncpsb.org/batman-tcm/) [12, 13]. During the screening for active ingredients, the absorption, distribution, metabolism, and excretion (ADME) of drug compounds were the essential limitations. Oral bioavailability (OB) and drug-likeness (DL) are two of the most crucial pharmacokinetic parameters in ADME processes [14]. OB refers to the speed and extent of absorption of an orally administered drug that enters the body’s circulation through the liver after absorption from the gastrointestinal tract. DL is defined as the structural similarity of an herbal ingredient to a known drug. Compounds with an OB≥30% and a DL≥0.18 were screened as bioactive ingredients in the current study [15, 16].

### YSTLF compound targets

The putative targets of the bioactive ingredients in YSTLF were obtained from the TCMSP database and the BATMAN-TCM database. The targets downloaded from the TCMSP database were converted from the protein name to the gene name via R language. All gene targets of YSTLF were retrieved after summarizing and deleting duplicates.

### Oligoasthenozoospermia gene targets

The keywords ‘asthenospermia’, ‘oligospermia’, ‘low sperm motility’, ‘spermatogenic dysfunction’ and ‘deficiency of sperm motility’ were used in the GeneCards database (https://www.genecards.org/) and the DisGeNET database (http://www.disgenet.org/) to collect oligoasthenozoospermia-related targets. The GeneCards database provides comprehensive, user-friendly information on all annotated and predicted human genes [17]. The DisGeNET database is a discovery platform containing one of the largest publicly available collections of genes and variants associated with human diseases [18]. The intersection between the putative target genes of YSTLF and oligoasthenozoospermia-related targets was determined with the online tool Venny 2.1 (http://bioinfogp.cnb.csic.es/tools/venny/) and these were considered the potential targets for the bioactive ingredients in YSTLF to treat oligoasthenozoospermia, which was visualized with a Venn diagram.

### Protein-protein interaction (PPI) network analysis

The common targets of YSTLF and oligoasthenozoospermia were put into the STRING database (https://string-db.org/, version 11.0) for PPI network analysis. The interacting proteins with a confidence score of ≥0.900 were chosen for PPI network visualization construction [19]. Then, the constructed PPI network was displayed in Cytoscape software (version 3.7.2) [20], which was based on the information from the STRING database. The CytoHubba plugin (version 1.0) of Cytoscape was applied to compute the top ten genes with a high degree [21]. It is generally believed that the node with the highest degree, highest betweenness centrality and highest closeness centrality is the most topologically important in the network. In other words, the top ten genes were potential hub targets.

### GO and KEGG pathway analyses

The Gene Ontology (GO) knowledgebase can provide information on the functions of genes, including biological processes (BPs), cellular components (CCs) and molecular functions (MFs) [22]. The Kyoto Encyclopedia of Genes and Genomes (KEGG) is a database resource for understanding high-level gene functions and genomic information [23]. GO and KEGG pathway enrichment analyses were conducted in Metascape (http://metascape.org/), a gene annotation and analysis resource [24]. According to a high count and P<0.05, the top 20 BP, CC, and MF GO terms and the top 20 KEGG pathways were selected for functional annotation clustering to construct enrichment analysis bubble diagrams by the clusterProfiler package in R [25]. Enrichment analyses could contribute to further probes of the biological functions and potential mechanisms of the YSTLF targets in oligoasthenozoospermia.

### Construction of the networks and analysis

To further clarify the molecular mechanism of YSTLF in oligoasthenozoospermia treatment, Cytoscape 3.7.2 was applied to establish a disease-formula-compound-target-pathway network [26]. In this graphical network, the disease, formula, compounds, targets and pathways were expressed as nodes, whereas the disease-formula-compound-target-pathway interactions were expressed as edges.

## Results

### YSTLF bioactive components

Based on the OB and DL values, 130 active components were finally obtained by searching the TCMSP database and the BATMAN-TCM database. Specifically, the numbers of candidate ingredients in TSZ, YYH, SDH, HQ, DS, CNX and TSZ were 11, 23, 2, 20, 65, 4, and 15, respectively. Among all ingredients, 7 involved common compounds from two or more drugs. For example, quercetin is a common compound in the four drugs TSZ, YYH, HQ, and CNX, and it has antioxidant, anti-inflammatory, antiviral, and antitumor activities and regulates glucose and lipid metabolism, and immune functions [27].

### Potential targets of YSTLF for oligoasthenozoospermia

Among the 130 candidate components, 955 targets were retrieved from the TCMSP database and the BATMAN-TCM database. Finally, a total of 250 ingredient targets in YSTLF were obtained after eliminating the overlapping targets. The numbers of potential targets connected by TSZ, YYH, SDH, HQ, DS, CNX and TSZ were 108, 106, 21, 100, 61, 88, and 142, respectively.

According to previous literature, oligoasthenozoospermia is a genetic disease. Based on the GeneCards database and the DisGeNET database, a total of 3677 genes associated with oligoasthenozoospermia were obtained after deleting duplicates. Specifically, the numbers associated with ‘asthenospermia’, ‘oligospermia’, ‘low sperm motility’, ‘spermatogenic dysfunction’ and ‘deficiency of sperm motility’ were 145, 672, 3229, 771, and 2936, respectively.

**Fig 2:**
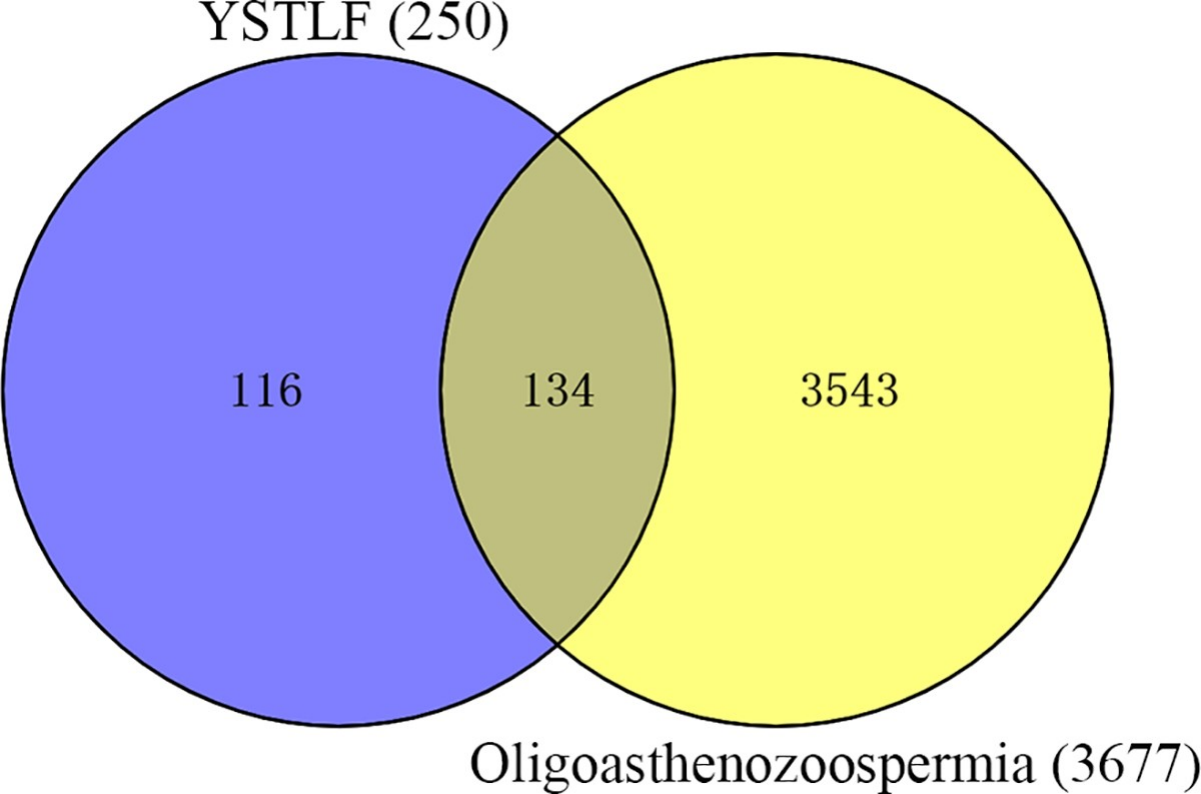
Venn diagram of the drugs and disease targets.

Then, the intersection between 250 YSTLF targets and 3677 oligoasthenozoospermia-related targets was determined through the online tool Venny 2.1. Consequently, a total of 134 potential targets associated with both oligoasthenozoospermia and YSTLF were identified with Venn diagrams (**Fig 2**).

### YSTLF compound-target network

**Fig 3:**
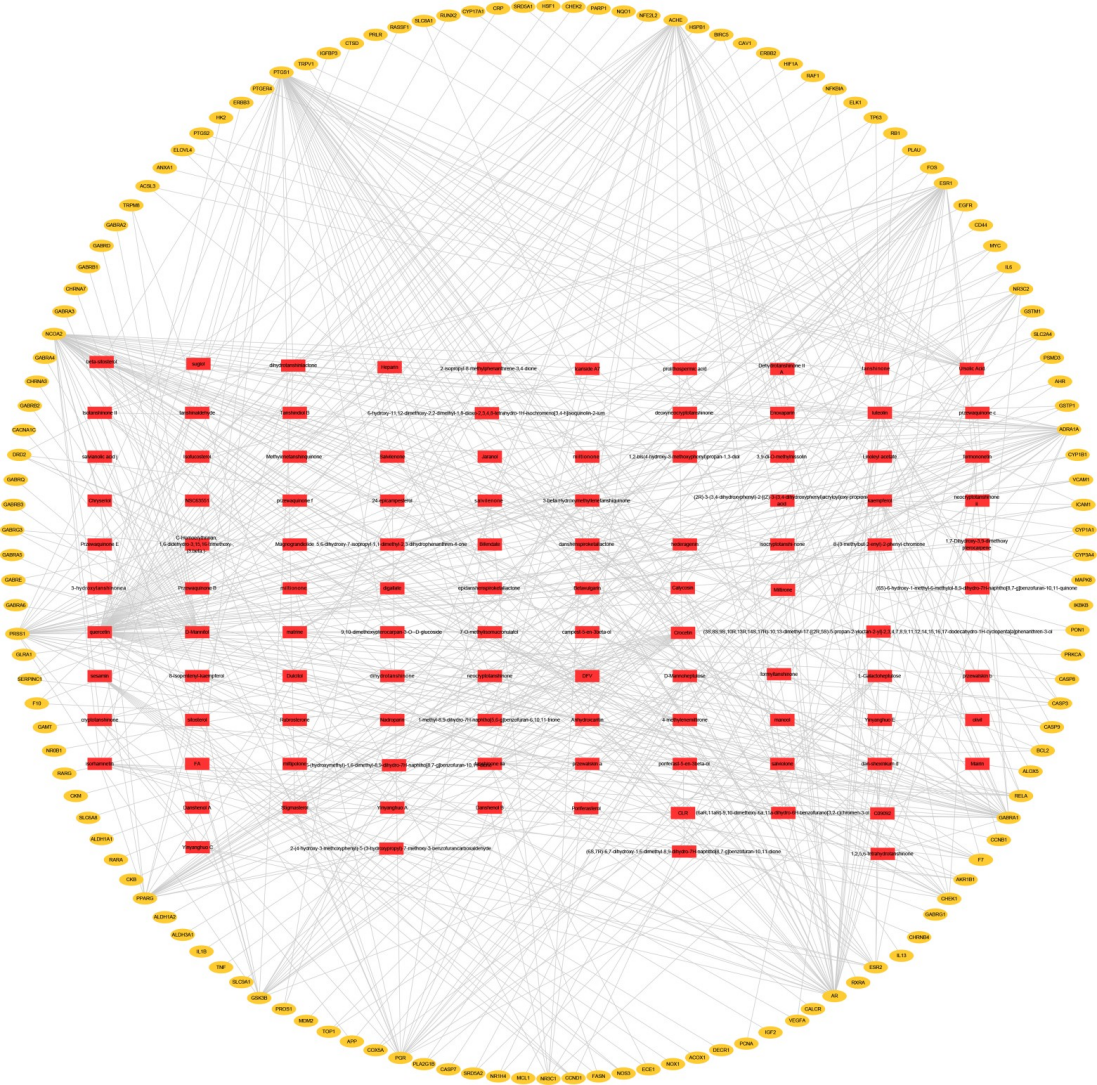
Compound-target network of YSTLF. Red rectangles represent the compounds in YSTLF, and orange circles represent the targets of oligoasthenozoospermia.

As Chinese medicinal formulae exhibit multiple pharmacological effects by interacting with multiple targets, it is of great significance for us to explore the underlying mechanisms of Chinese medicinal formulae on complex diseases through network analysis. Cytoscape 3.7.2 was used to construct the compound-target network of YSTLF on oligoasthenozoospermia (**Fig 3**). Through network analysis in Cytoscape 3.7.2, there were 240 nodes (106 for candidate bioactive ingredients and 134 for potential targets) as well as 607 edges in the network. Based on the topological analysis of the network, the number of edges or targets related to the nodes is taken as the degree value. Among all the bioactive ingredients, the top 10 with the highest degree of nodes were quercetin, luteolin, kaempferol, crocetin, D-mannitol, ursolic acid, tanshinone iia, isorhamnetin, anhydroicaritin, and beta-sitosterol (**Table 1**), which were involved in the regulation of multiple oligoasthenozoospermia targets. The top 10 compounds mentioned above could be regarded as potentially the most bioactive core components. These findings may provide new mechanisms for the treatment of oligoasthenozoospermia by YSTLF.

**Table 1:** Top 10 bioactive ingredients with a high degree.

Mol ID	Molecule Name	Degree
MOL000098	quercetin	59
MOL000006	luteolin	26
MOL000422	kaempferol	25
MOL001406	crocetin	22
MOL000003	D-mannitol	21
MOL000511	ursolic acid	17
MOL007154	tanshinone iia	14
MOL000354	isorhamnetin	14
MOL004373	anhydroicaritin	11
MOL000358	beta-sitosterol	11

### PPI network

**Fig 4:**
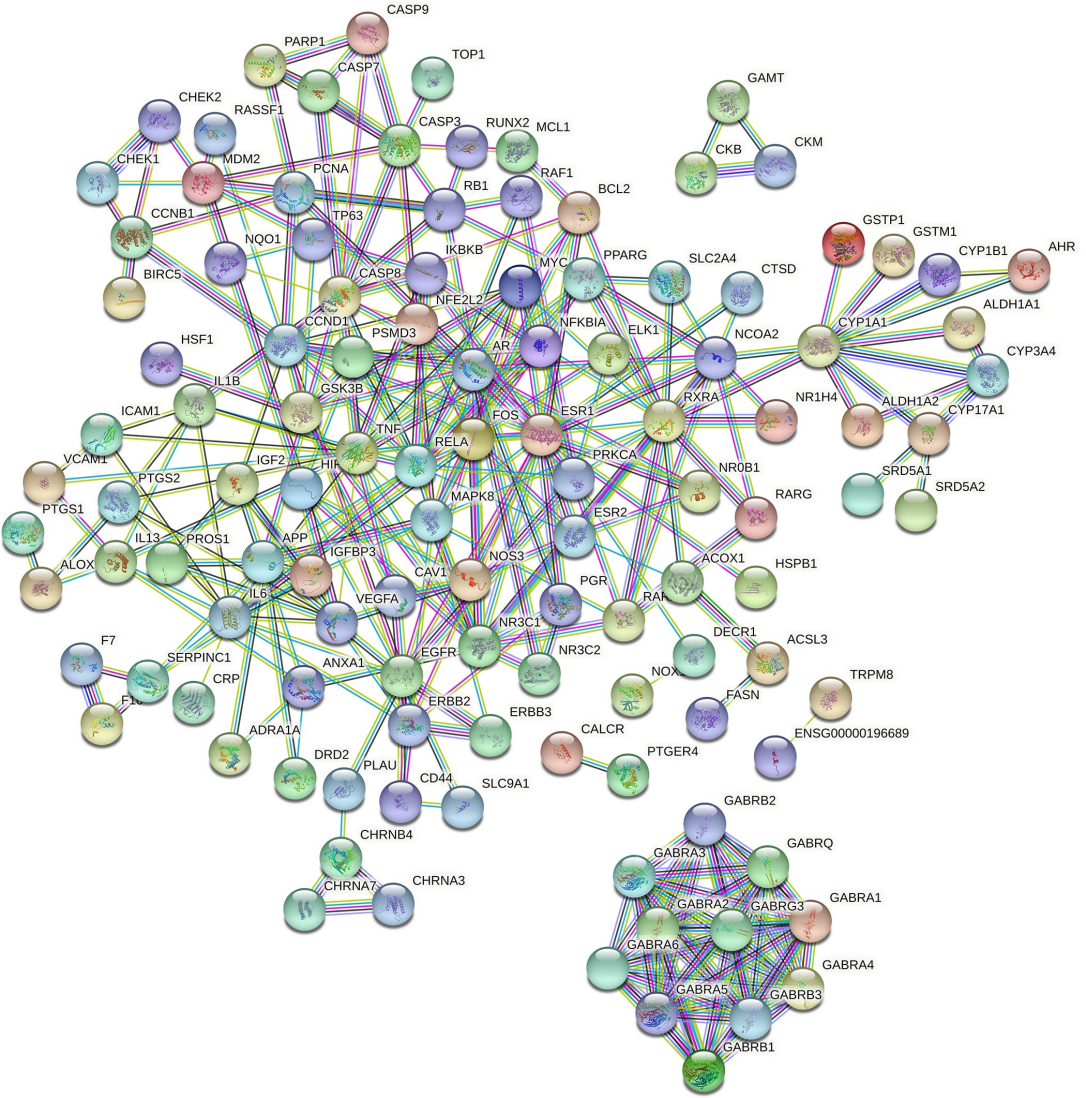
Common target protein interaction network.

**Fig 5:**
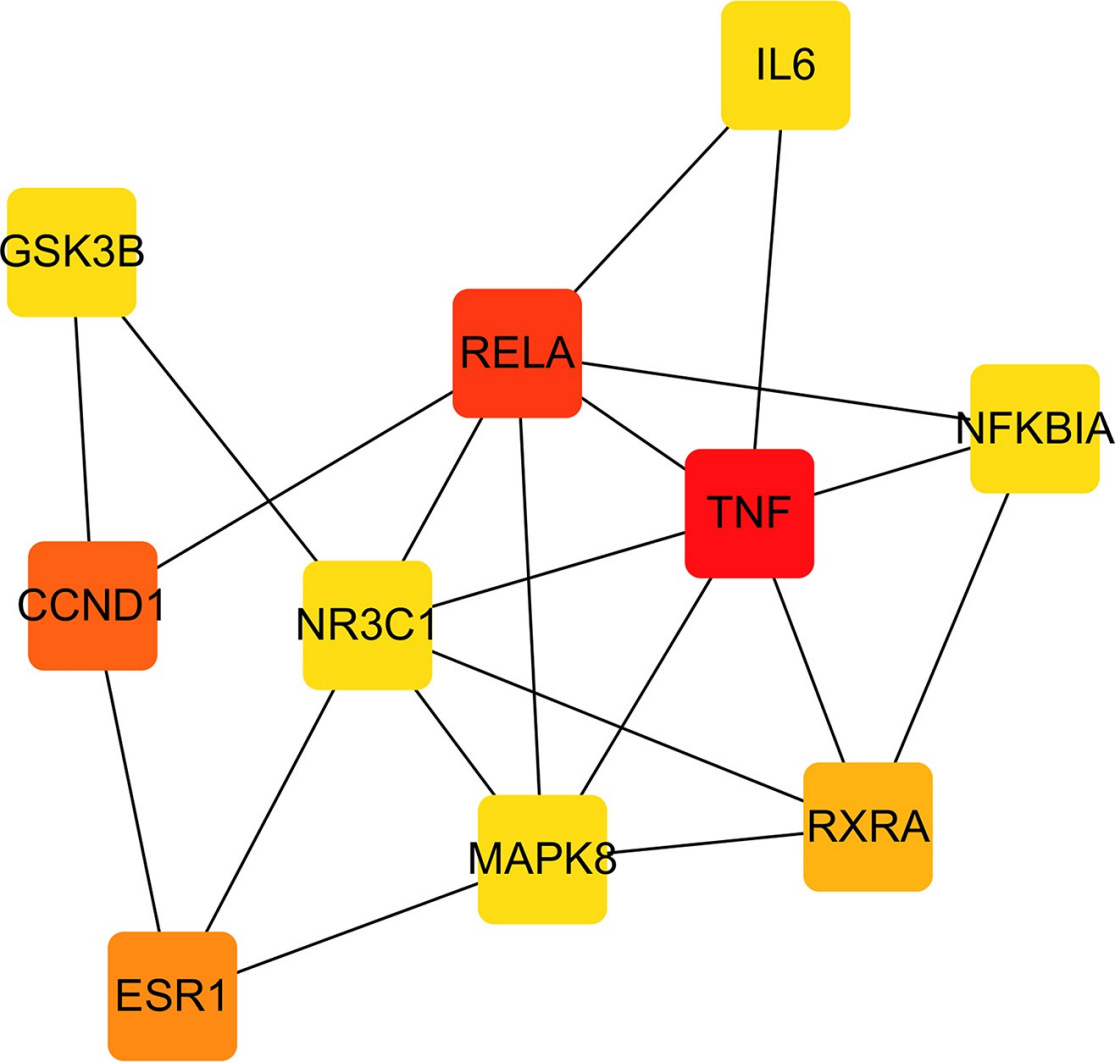
Analysis of the top 10 hub gene networks of YSTLF for oligoasthenozoospermia treatment by the MCC algorithm, in which red and yellow represent the importance in the network.

Growing studies have confirmed that diseases are not caused by only a single gene but also via interactions among multiple targets. To further elaborate the molecular mechanisms of the pharmacological actions of YSTLF on oligoasthenozoospermia, 134 potential targets were input into the STRING database to construct a PPI network. In the network, the nodes and edges represent proteins and protein-protein associations, respectively. In the current study, 134 nodes and 580 edges were established in the network after hiding disconnected nodes (**Fig 4**). The average node degree and the average local clustering coefficient were 8.66 and 0.541, respectively. The top 10 hub genes (i.e., TNF, RELA, CCND1, ESR1, RXRA, NFKBIA, GSK3B, NR3C1, MAPK8, IL6), which were considered potential hub targets of YSTLF for treating oligoasthenozoospermia, were identified through the CytoHubba plugin with maximal clique centrality (MCC) according to their degree (**Fig 5**). The network characteristics of the potential hub targets are presented in **Table 2**.

**Table 2:** The PPI network characteristics of the top 10 hub targets.

Target	Betweenness	Closeness	Degree
TNF	1556.31697	58.56667	26
RELA	1260.83584	57.63333	24
CCND1	1124.66339	54.35	19
ESR1	1052.45654	52.48333	16
RXRA	2025.27701	53.31667	15
NFKBIA	220.14611	49.31667	14
GSK3B	392.20756	47.13333	14
NR3C1	658.08329	52.4	14
MAPK8	992.50018	54.06667	14
IL6	851.07865	50.06667	14

Closeness and betweenness are two more important parameters in the network. Closeness refers to the reciprocal of the average length of the shortest circuit between a node and all other nodes in the network. The higher the value is, the greater the centrality of the node, indicating that the transmission speed of the signal from one node to the other nodes is faster. Betweenness is defined as the shortest path of all node pairs in the network, and the shorter the paths through a node are, the more important that node is.

### GO terms enrichment analysis

**Fig 6:**
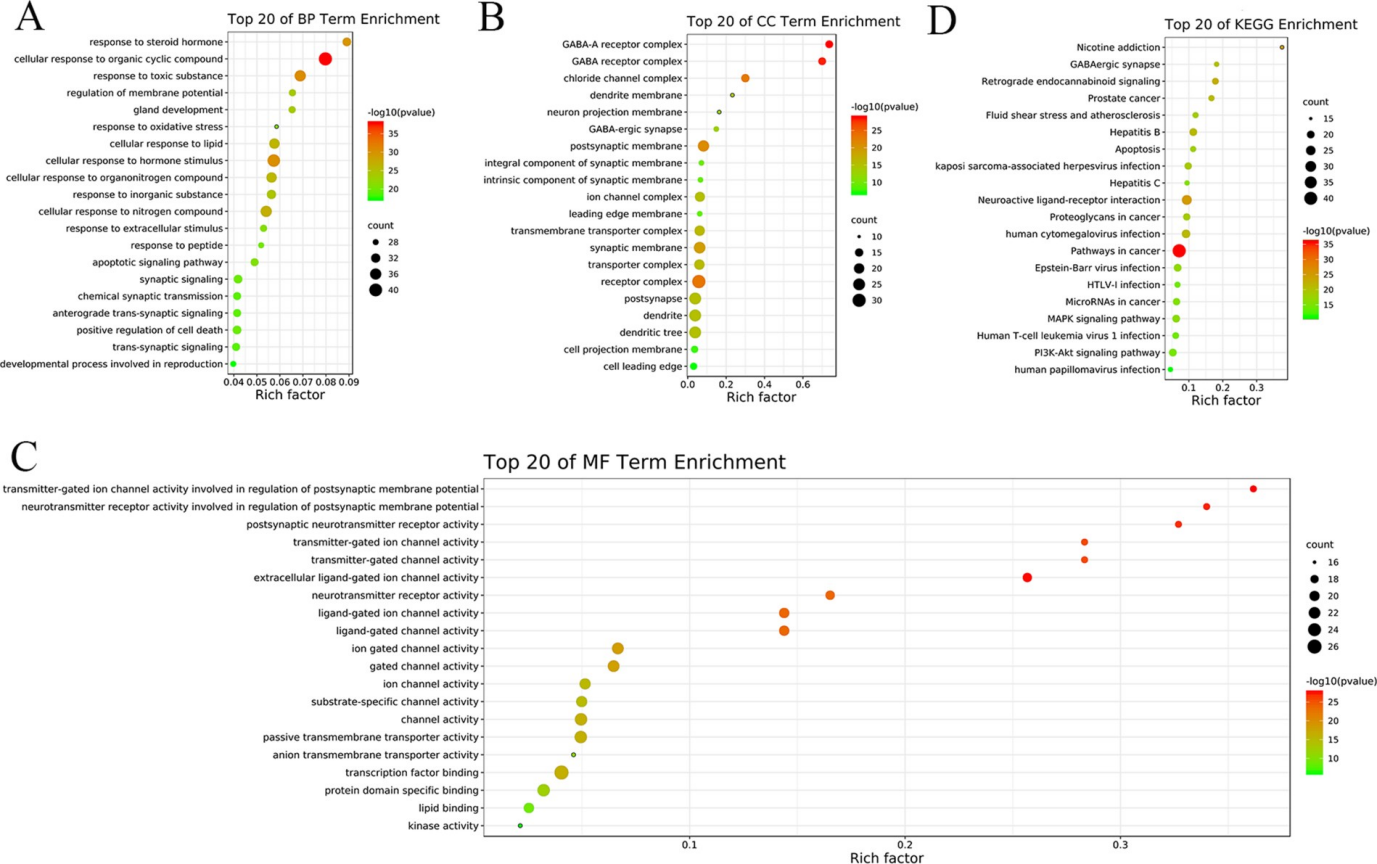
GO and KEGG analysis of targets. (A) GO biological process terms. (B) GO cellular component terms. (C) GO molecular function terms. (D) KEGG pathway. The size of each dot corresponds to the number of genes annotated in the entry, and the color of each dot corresponds to the corrected p-value.

To illustrate the multiple biological functions of potential targets in oligoasthenozoospermia with treatment by YSTLF, the above common targets were entered into Metascape for GO enrichment analysis. The results of the GO analysis showed that the biological process (BP) was significantly enriched in the cellular response to organic cyclic compounds (GO: 0071407), cellular response to hormone stimuli (GO: 0032870), response to toxic substances (GO: 0009636), cellular response to nitrogenous compounds (GO: 1901699), cellular response to lipids (GO:0071396) (**Fig 6A**), cellular components (CCs) in receptor complexes (GO: 0043235), postsynaptic (GO: 0098794), dendrites (GO: 0030425), dendritic tree (GO: 0097447), synaptic membrane (GO: 0097060) (**Fig 6B**), molecular function (MF) in transcription factor binding (GO: 0008134), channel activity (GO: 0015267), passive transmembrane transporter activity (GO: 0022803), protein domain-specific binding (GO: 0019904), and ion gated channel activity (GO: 0022839) (**Fig 6C**). According to the count, the top 10 BPs, top 10 CCs, and top 10 MFs are presented in **Table 3**.

**Table 3:** GO enrichment analysis.

Category	Term	Count	P value
	GO:0071407~cellular response to organic cyclic compound	41	1.44229E-38
	GO:0032870~cellular response to hormone stimulus	39	4.01273E-31
	GO:0009636~response to toxic substance	36	1.8724E-31
	GO:1901699~cellular response to nitrogen compound	36	8.96277E-28
BP	GO:0071396~cellular response to lipid	34	4.05399E-27
	GO:0071417~cellular response to organonitrogen compound	34	8.29749E-27
	GO:0010035~response to inorganic substance	32	3.30251E-25
	GO:0048545~response to steroid hormone	31	1.71101E-30
	GO:0099536~synaptic signaling	31	1.28938E-20
	GO:0010942~positive regulation of cell death	31	1.75892E-20
	GO:0043235~receptor complex	30	5.57143E-24
	GO:0098794~post synapse	25	5.3657E-16
	GO:0030425~dendrite	25	5.76475E-16
	GO:0097447~dendritic tree	25	6.19191E-16
CC	GO:0097060~synaptic membrane	24	5.88958E-20
	GO:0045211~postsynaptic membrane	23	6.55646E-22
	GO:1902495~transmembrane transporter complex	20	8.97726E-17
	GO:1990351~transporter complex	20	1.44266E-16
	GO:0034702~ion channel complex	19	3.68031E-07
	GO:0034707~chloride channel complex	15	1.38207E-23
	GO:0008134~transcription factor binding	26	5.25479E-17
	GO:0015267~channel activity	23	5.2142E-17
	GO:0022803~passive transmembrane transporter activity	23	5.46442E-17
	GO:0019904~protein domain specific binding	23	4.86877E-13
MF	GO:0022839~ion gated channel activity	22	5.04078E-19
	GO:0022836~gated channel activity	22	9.55578E-19
	GO:0005216~ion channel activity	21	6.33001E-16
	GO:0022838~substrate-specific channel activity	21	1.18187E-15
	GO:0008289~lipid binding	20	1.21686E-09
	GO:0015276~ligand-gated ion channel activity	20	4.02787E-24

### KEGG pathway enrichment analysis

To elucidate the crucial signaling pathways of YSTLF in the treatment of oligoasthenozoospermia, the 134 potential targets were input into Metascape for KEGG pathway enrichment analysis. The top 20 pathways were screened based on the parameters of counts as well as in combination with P-values (**Table 4**), including the MAPK signaling pathway (hsa04010), PI3K/Akt signaling pathway (hsa04151), and apoptosis (hsa04210). Excluding the above, the corresponding common signaling pathways also focused on the TNF signaling pathway (hsa04668), AGE-RAGE signaling pathway in diabetic complications (hsa04933), and IL-17 signaling pathway (hsa04657), which had lower P-values. The KEGG pathways were visualized via the Omcishare website (https://www.omicshare.com/) based on their corresponding counts (**Fig 6D**).

**Table 4:** KEGG pathway enrichment analysis based on the YSTLF-oligoasthenozoospermia network (top 20 with count).

Pathway	Genes	Count	P value
Pathways in cancer	AKR1B1, BIRC5, AR, CCND1, BCL2, CASP3, CASP7, CASP8, CASP9, NQO1, EGFR, ELK1, ERBB2, ESR1, ESR2, FASN, FOS, GSK3B, GSTM1, GSTP1, HIF1A, IGF2, IKBKB, IL6, IL13, MDM2, MYC, NFE2L2, NFKBIA, PPARG, PRKCA, MAPK8, PTGER4, PTGS2, RAF1, RARA, RB1, RELA, RXRA, VEGFA, RASSF1	41	1.3986E-36
Neuroactive ligand-receptor interaction	ADRA1A, CALCR, CHRNA3, CHRNA7, CHRNB4, DRD2, GABRA1, GABRA2, GABRA3, GABRA4, GABRA5, GABRA6, GABRB1, GABRB2, GABRB3, GABRD, GABRE, GABRG1, GABRG3, GLRA1, NR3C1, PRLR, PRSS1, PTGER4, TRPV1, GABRQ	26	3.3424E-26
human cytomegalovirus infection	CCND1, CASP3, CASP8, CASP9, EGFR, ELK1, FASN, GSK3B, IKBKB, IL1B, IL6, MDM2, MYC, NFKBIA, PRKCA, PTGER4, PTGS2, RAF1, RB1, RELA, TNF, VEGFA	22	4.9049E-22
Hepatitis B	BIRC5, CCND1, BCL2, CASP3, CASP8, CASP9, ELK1, FASN, FOS, IKBKB, IL6, MYC, NFKBIA, PCNA, PRKCA, MAPK8, RAF1, RB1, RELA, TNF	20	6.0125E-22
Epstein-Barr virus infection	CCND1, BCL2, CASP3, CASP8, CASP9, CD44, FASN, GSK3B, HSPB1, ICAM1, IKBKB, IL6, MDM2, MYC, NFKBIA, MAPK8, PSMD3, RB1, RELA, TNF	20	2.1281E-17
MAPK signaling pathway	CACNA1C, CASP3, EGFR, ELK1, ERBB2, ERBB3, FASN, FOS, HSPB1, IGF2, IKBKB, IL1B, IL6, MYC, PRKCA, MAPK8, RAF1, RELA, TNF, VEGFA	20	7.9572E-17
PI3K-Akt signaling pathway	CCND1, BCL2, CASP9, EGFR, ERBB2, ERBB3, GSK3B, IGF2, IKBKB, IL6, MCL1, MDM2, MYC, NOS3, PRKCA, PRLR, RAF1, RELA, RXRA, VEGFA	20	2.1556E-15
kaposi sarcoma-associated herpesvirus infection	CCND1, CASP3, CASP8, CASP9, FASN, FOS, GSK3B, HIF1A, ICAM1, IKBKB, IL6, MYC, NFKBIA, MAPK8, PTGS2, RAF1, RB1, RELA, VEGFA	19	1.0748E-19
Proteoglycans in cancer	CCND1, CASP3, CAV1, CD44, EGFR, ELK1, ERBB2, ERBB3, ESR1, HIF1A, IGF2, MDM2, MYC, PLAU, PRKCA, RAF1, SLC9A1, TNF, VEGFA	19	2.5601E-19
MicroRNAs in cancer	CCND1, BCL2, CASP3, CD44, CYP1B1, EGFR, ERBB2, ERBB3, IKBKB, MCL1, MDM2, MYC, PLAU, PRKCA, PTGS2, RAF1, VEGFA, TP63, RASSF1	19	3.659E-16
Retrograde endocannabinoid signaling	CACNA1C, GABRA1, GABRA2, GABRA3, GABRA4, GABRA5, GABRA6, GABRB1, GABRB2, GABRB3, GABRD, GABRE, GABRG1, GABRG3, PRKCA, MAPK8, PTGS2, GABRQ	18	1.4255E-23
Human T-cell leukemia virus 1 infection	CCND1, CHEK1, ELK1, FOS, GSK3B, ICAM1, IKBKB, IL6, MYC, NFKBIA, PCNA, PRKCA, MAPK8, RB1, RELA, TNF, VCAM1, CHEK2	18	4.6747E-15
Prostate cancer	AKR1B1, AR, CCND1, BCL2, CASP9, EGFR, ERBB2, GSK3B, GSTP1, IKBKB, MDM2, NFKBIA, PLAU, RAF1, RB1, RELA, SRD5A2	17	8.6806E-22
Fluid shear stress and atherosclerosis	BCL2, CAV1, NQO1, FOS, GSTM1, GSTP1, ICAM1, IKBKB, IL1B, NFE2L2, NOS3, MAPK8, RELA, TNF, VCAM1, VEGFA, NOX1	17	3.1072E-19
Apoptosis	PARP1, BIRC5, BCL2, CASP3, CASP7, CASP8, CASP9, CTSD, FASN, FOS, IKBKB, MCL1, NFKBIA, MAPK8, RAF1, RELA, TNF	17	9.0654E-19
HTLV-I infection	CCND1, CHEK1, ELK1, FOS, GSK3B, ICAM1, IKBKB, IL6, MYC, NFKBIA, PCNA, MAPK8, RB1, RELA, TNF, VCAM1, CHEK2	17	6.9835E-15
GABAergic synapse	CACNA1C, GABRA1, GABRA2, GABRA3, GABRA4, GABRA5, GABRA6, GABRB1, GABRB2, GABRB3, GABRD, GABRE, GABRG1, GABRG3, PRKCA, GABRQ	16	3.265E-21
Hepatitis C	CCND1, CASP3, CASP8, CASP9, EGFR, FASN, GSK3B, IKBKB, MYC, NFKBIA, MAPK8, RAF1, RB1, RELA, RXRA, TNF	16	1.6607E-16
human papillomavirus infection	CCND1, CASP3, CASP8, EGFR, FASN, GSK3B, IKBKB, MDM2, PRKCA, PTGER4, PTGS2, RAF1, RB1, RELA, TNF, VEGFA	16	1.4763E-11
Nicotine addiction	CHRNA7, GABRA1, GABRA2, GABRA3, GABRA4, GABRA5, GABRA6, GABRB1, GABRB2, GABRB3, GABRD, GABRE, GABRG1, GABRG3, GABRQ	15	2.5707E-25

**Fig 7:**
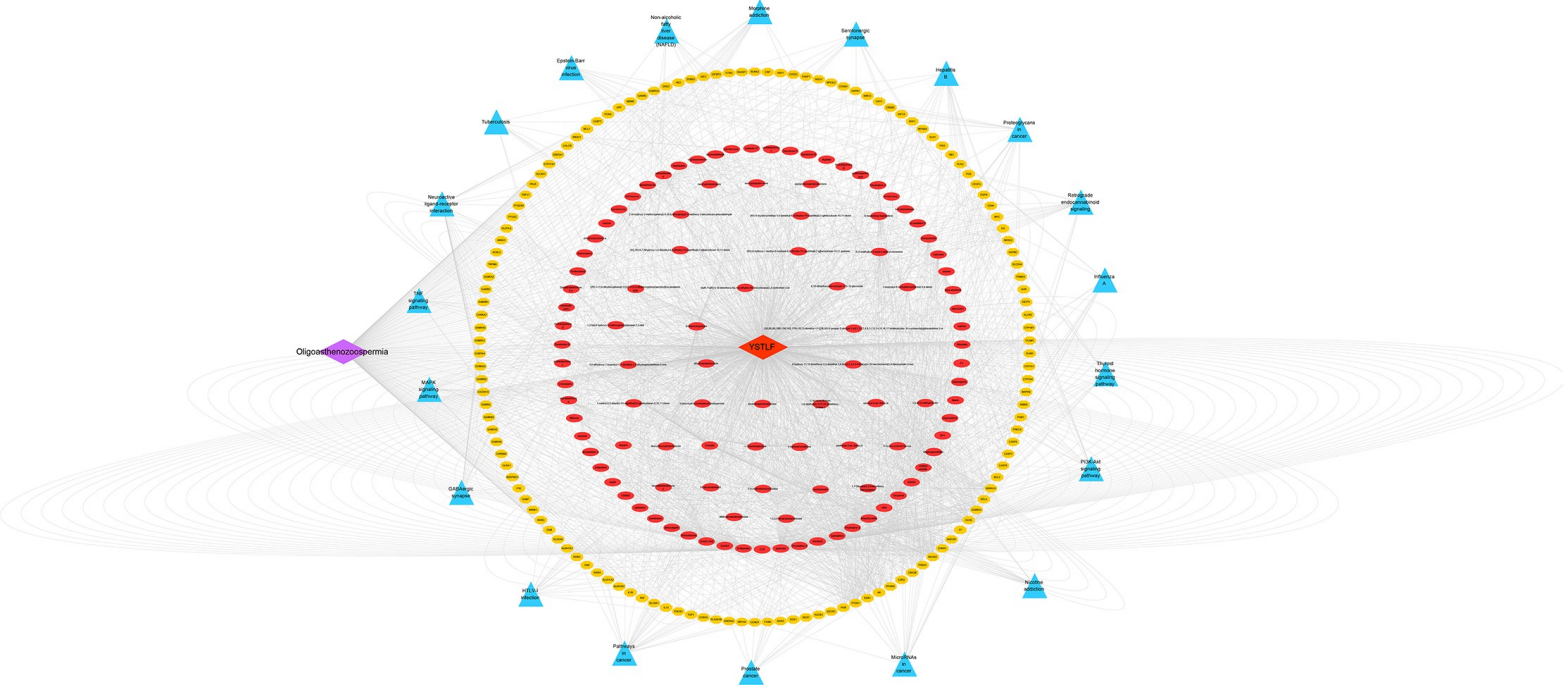
Disease-formula-compound-target-pathway network. Purple rhombuses represent disease, red rhombuses represent formulae, red ellipses represent the chemical compounds in YSTLF related to the common targets, yellow polygons represent the common targets from the chemical compounds and oligoasthenozoospermia, and blue triangles represent the main biological pathways.

**Fig 8:**
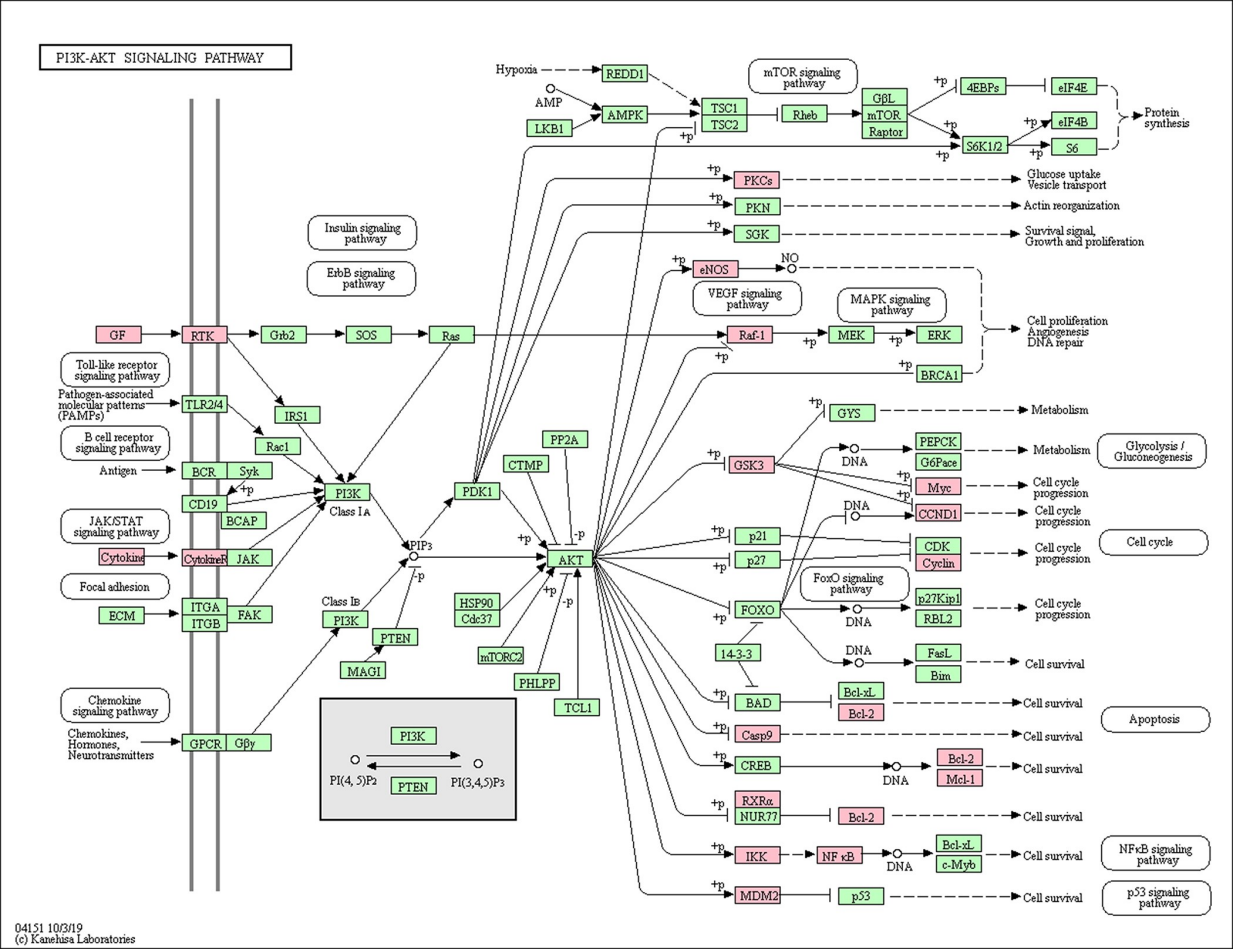
PI3K-AKT signaling pathway. The red rectangle represents the targets related to the core component-target-pathway network.

**Fig 9:**
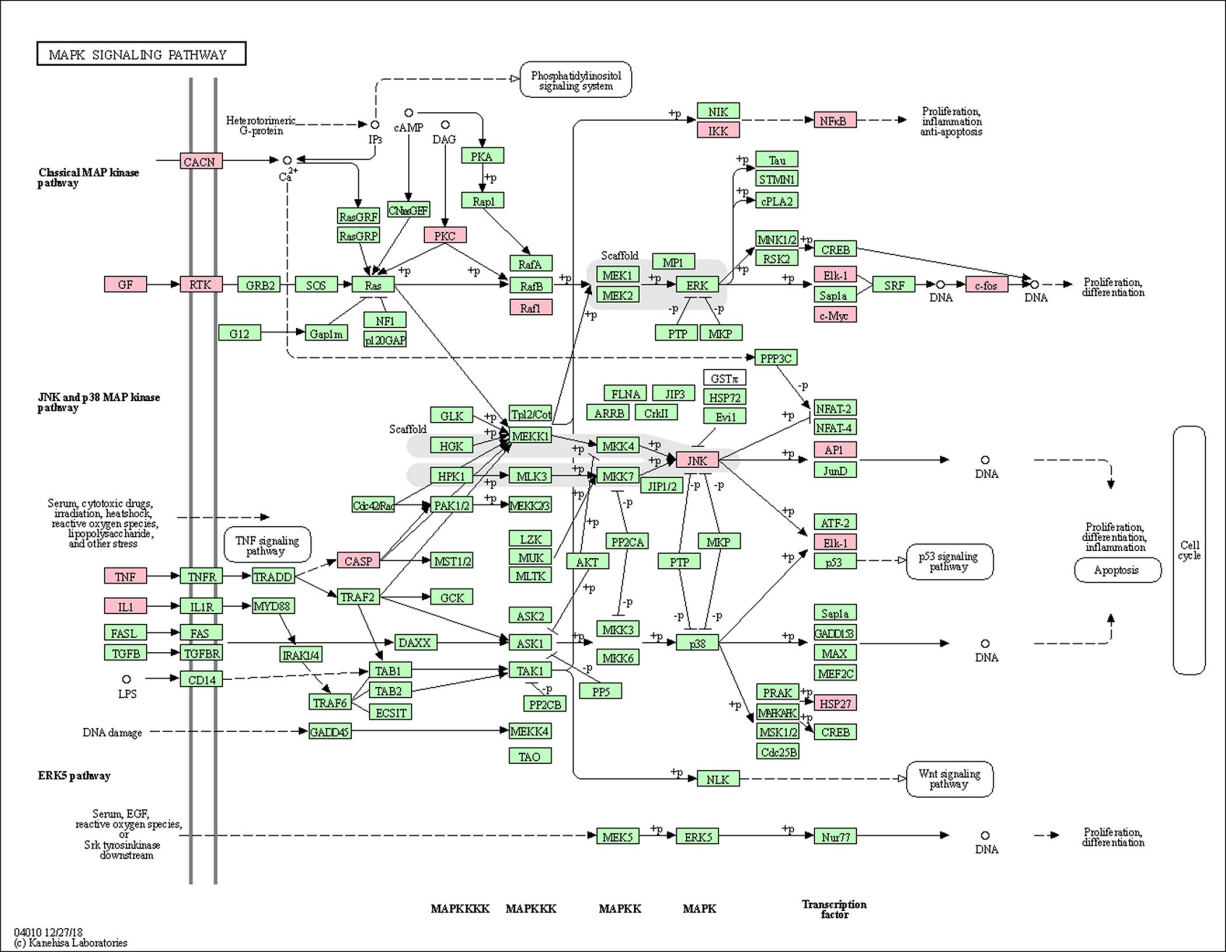
MAPK signaling pathway. The red rectangle represents the targets related to the core component-target-pathway network.

**Fig 10:**
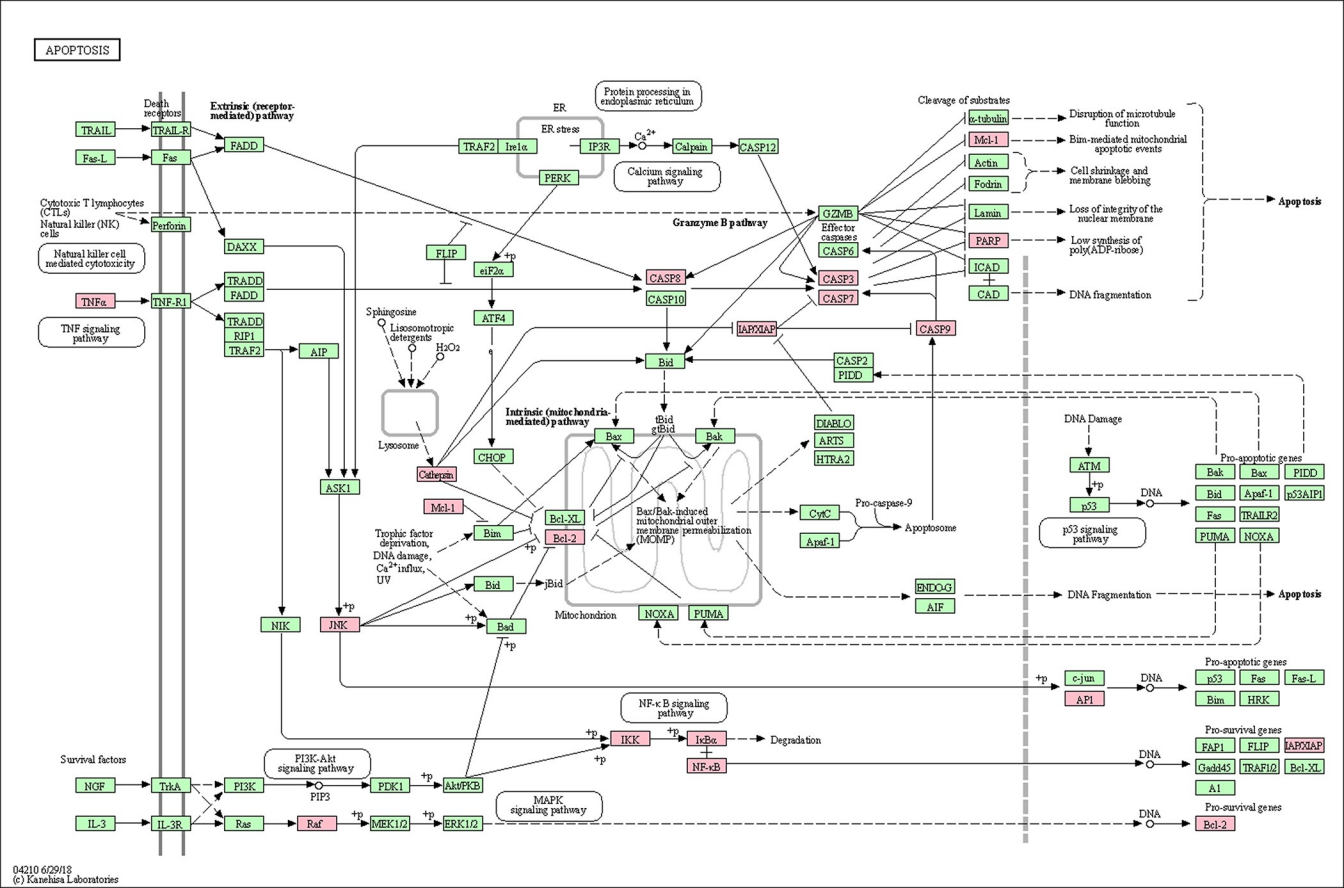
Apoptosis signaling pathway. The red rectangle represents the targets related to the core component-target-pathway network.

A disease-formula-compound-target-pathway network was constructed using the Cytoscape 3.7.2 platform (**Fig 7**). Based on the above results, the PI3K/Akt, MAPK and apoptosis signaling pathways were found to be the most important signaling pathways during the process of treating oligoasthenozoospermia with YSTLF. The targets related to these three signaling pathways are shown in Figs 8–10, which were retrieved from the KEGG database (http://www.kegg.jp/kegg/mapper.html).

## Discussion

Oligoasthenozoospermia is a multifactorial disease that is associated with a variety of proteins or pathways during its occurrence and progression, such as calcium- and integrin-binding protein-1 (CIB1) and the TGF-β1/Smad signaling pathway [28, 29]. On account of their multicomponent compositions, Chinese herbal medicines may exhibit a wide range of pharmacological activities with the characteristics of multiple targets and multiple pathways, which may be beneficial to the treatment of oligoasthenozoospermia [30]. YSTLF has achieved satisfactory therapeutic effects in the treatment of oligoasthenozoospermia. According to the literature, YSTLF with or without minimally invasive surgery in the treatment of varicocele infertility could improve the sperm motility rate, sperm linear motion velocity, sperm concentration, sperm DNA integrity and spousal pregnancy rate [6, 31]. However, the underlying mechanisms of YSTLF in the treatment of oligoasthenozoospermia remain unclear and need to be elucidated through an in-depth systematic study at the molecular level. Based on the theories of molecular biology, systems biology and pharmacology, network pharmacology can construct intricate interaction networks according to the bioactive compounds, target molecules, and biological functions and may clarify the potential molecular mechanisms of complicated Chinese medicine (CM) formulae in diseases. The network pharmacology approach may be a promising systematic mechanistic research strategy for CM formula studies. Thus, in the current study, the above approach was adopted to illuminate the underlying pharmacological mechanisms of YSTLF in oligoasthenozoospermia treatment. The components in YSTLF with OB≥30% and DL≥0.18 were deemed pharmacokinetically active and are probably absorbed and distributed in the body. Accordingly, the 106 bioactive ingredients (the top 10 compounds with high degrees were quercetin, luteolin, kaempferol, crocetin, D-mannitol, ursolic acid, tanshinone iia, isorhamnetin, anhydroicaritin and beta-sitosterol) and the 134 potential targets (the 10 hub targets were TNF, RELA, CCND1, ESR1, RXRA, NFKBIA, GSK3B, NR3C1, MAPK8, IL6) of YSTLF for oligoasthenozoospermia treatment were predicted, which could likely reveal the potential pharmacological mechanisms of YSTLF.

According to the literature, factors affecting the count and quality of sperm include oxidative stress, inflammatory reactions, immunologic derangement and DNA damage to sperm, etc. In this study, quercetin was the most significant compound, which is closely related to semen quality. Quercetin, a compound belonging to the flavonol class, has extensive pharmacological actions, such as antioxidant and anti-inflammatory effects [32]. In vitro experiments have shown that adding quercetin to semen can elevate the total antioxidant potential and ameliorate lipid peroxidation during cryopreservation to improve sperm motility, acrosome integrity, plasma membrane integrity, mitochondrial activity and chromatin condensation [33]. Diao et al. demonstrated that quercetin can significantly boost sperm motility in leukocytospermic patients, and the mechanism could be that quercetin can reduce the levels of HO and the content of mtDNA and increase the contents of CytB and NADH5 in sperm to display intensive antioxidant activity against ROS-mediated sperm damage [34]. In addition, luteolin and kaempferol, which are also flavonoids with antioxidant effects, can improve sperm quality by increasing the activity of superoxide dismutase (SOD) and glutathione peroxidase (GPx) or repairing sperm DNA damage [35, 36]. Crocetin, a carotenoid, also has strong antioxidant activity, especially in the suppression of lipid peroxidation reactions; on the other hand, it can decrease testicular apoptosis by reducing caspase 3 activity, thus promoting spermatogenic function [37]. Isorhamnetin-containing flavonoids and the phytosterol derivative beta-sitosterol can both alleviate oxidative stress and apoptosis in sperm [38, 39]. A network pharmacology approach together with all of these studies can predict that YSTLF may improve the count and quality of sperm by exerting antioxidant and antiapoptotic effects, thus improving male fertility.

Among the top 10 hub targets, many genes were found to be involved in spermatogenesis, sperm motility and sperm morphology, which play an important role in the therapeutic action of YSTLF for oligoasthenozoospermia treatment. As inflammatory cytokines, increased TNF and IL6 in the male reproductive tract could jeopardize spermatogenesis as well as the functions of sperm and ejaculation, which are closely related to fertility problems [40, 41]. In addition, TNF and IL6 could activate apoptotic mechanisms in human spermatozoa [42–44]. As an inflammatory mediator, the content of NFKBIA may decrease, while cytokines such as IL6 may increase in the epididymitis. The above early inflammatory signaling events could adversely affect male fertility [45]. Cyclin D1 (CCND1), a cell cycle factor, is associated with cell cycle progression and spermatogenesis [46]. ESR1 is expressed throughout the reproductive tracts of adult male mice, and mutation to ESR1 can reduce sperm viability without affecting testicular size or sperm count, thus impairing male fertility [47]. Furthermore, polymorphisms in exon 4 (LBD) of ESR1 also have a close association with male infertility [48]. Another study reported that ESR1 is essential for sperm survival and maturation during epididymal storage because it is involved in the reabsorption of fluid in the cavity during sperm transport from the testis to the epididymis head. In addition, ESR1 knockout causes reduced sperm content in the epididymis and decreased sperm motility [49]. The NR3C1 protein, which is mainly distributed in peritubular cells, Sertoli cells, Leydig cells and spermatogonia of the adult testis, has been shown to be strongly associated with sperm motility [50]. The C‑Jun N‑terminal kinase MAPK8, which is a key gene in the Aktsignaling pathway, is considered to be associated with sperm apoptosis [51]. Based on the above results, YSTLF may increase sperm count and improve sperm motility by regulating cell proliferation, survival, and apoptosis and the inflammatory response of reproduction-related cells in males.

In the present study, KEGG pathway enrichment analysis showed that there were multiple signaling pathways involved in the treatment of oligoasthenozoospermia with YSTLF. Among them, the PI3K-Akt, MAPK and apoptosis pathways, in which YSTLF-associated hub targets were enriched, are closely related to oligoasthenozoospermia. The PI3K/Akt signaling pathway can participate in many important processes of male reproduction, such as the proliferation, differentiation, growth and apoptosis of spermatogonia and regulation of the hypothalamic-pituitary-gonad (HPG) axis during spermatogenesis [52]. Hypoxia and oxidative stress can inhibit the PI3K/Akt signaling pathway, reducing the level of Akt phosphorylation and inducing cell apoptosis [53]. Other studies have demonstrated that activation of the PI3K/Akt pathway may protect testicular cells from apoptosis in the offspring of mice after direct maternal exposure to di-(2-ethylhexyl) phthalate (DEHP) [52, 54]. In another study, suppression of the PI3K/Akt pathway associated with oxidative stress may lead to aflatoxin B_1_ (AFB_1_)-induced testicular damage and promote autophagy [55]. MAPK, one of the members of the serine/threonine protein kinase family, is involved in many physiological processes, such as cell proliferation, differentiation and growth, and P38, JNK and ERK, three protein genes in the MAPK pathway, are closely related to oligoasthenozoospermia [56]. The JNK and ERK1/2 MAPK signaling pathways, which are activated by di-N-butyl-phthalate (DBP), result in testicular injury, thus reducing sperm count by increasing apoptosis [57]. In addition, the P38 MAPK pathway could be activated by an abnormal arachidonic acid (AA) metabolic network, leading to decreased sperm motility [58]. Apoptosis plays a crucial role in spermatogenesis, so this pathway was predicted to be involved in the current study by the network pharmacology approach. NFKBIA, MAPK8, RELA, and TNF in the hub targets were enriched in the apoptosis signaling pathway. In summary, YSTLF may have therapeutic action in oligoasthenozoospermia mainly through antioxidative stress, antiapoptosis and anti-inflammation effects, which is basically consistent with the relevant research results of oligoasthenozoospermia in recent years. For instance, Morinda offcinalis–Lycium barbarum coupled-herbs (MOLBCH) can regulate the PI3K/Akt signaling pathway, prostate cancer, and the AGE-RAGE signaling pathway, regarded as the most representative pathways, by the core potential targets, the androgen receptor (AR), estrogen receptor (ESR1), mitogen-activated protein kinase 3 (MAPK3), RAC-alpha serine/threonine-protein kinase (AKT1), and glyceraldehyde-3-phosphate dehydrogenase (GAPDH) to alleviate apoptosis, promote male reproductive function, and reduce oxidant stress in the treatment of oligoasthenozoospermia [59].

However, the current study had some limitations. For instance, whether the functions of the pathways in this research were downregulated or upregulated was not clear. The potential targets of YSTLF in oligoasthenozoospermia treatment identified by the network pharmacology approach are only theoretical predictions and need to be verified by clinical and cell or animal experiments.

## Conclusions

In this study, the molecular mechanisms of YSTLF in oligoasthenozoospermia treatment were investigated by establishing multiple network models from a holistic viewpoint. It was demonstrated that the most effective compounds of YSTLF in the treatment of oligoasthenozoospermia may be quercetin, luteolin, kaempferol, crocetin, isorhamnetin and beta-sitosterol. The potential pharmacological actions of the above bioactive ingredients were closely related to antioxidative stress, antiapoptosis and anti-inflammation, with TNF, CCND1, ESR1, NFKBIA, NR3C1, MAPK8, and IL6 being possible targets. Furthermore, YSTLF may increase sperm count and motility mainly through the regulation of the PI3K/Akt, MAPK and apoptosis signaling pathways. The above study also suggested that network pharmacology predictions may be helpful tools to illustrate the molecular mechanisms of the Chinese herbal compound YSTLF in oligoasthenozoospermia treatment.

## References

[1] Kumar N, Singh AJJohrs. Trends of male factor infertility, an important cause of infertility: A review of literature. 2015;8(4):191–6. doi: 10.4103/0974-1208.170370 . 26752853 PMC4691969

[2] Agarwal A, Mulgund A, Hamada A, Chyatte MJRb, RB e. A unique view on male infertility around the globe. 2015;13:37. doi: 10.1186/s12958-015-0032-1 . 25928197 PMC4424520

[3] Min K, Min JJEp. Exposure to environmental noise and risk for male infertility: A population-based cohort study. 2017;226:118–24. doi: 10.1016/j.envpol.2017.03.069 . 28411496

[4] Sapozhkova Z, Eremin KJKld. [Updates in protocol for human semen examination.]. 2020;65(2):106–10. doi: 10.18821/0869-2084-2020-65-2-106-110 . 32159308

[5] Dyer S, Chambers G, de Mouzon J, Nygren K, Zegers-Hochschild F, Mansour R, et al. International Committee for Monitoring Assisted Reproductive Technologies world report: Assisted Reproductive Technology 2008, 2009 and 2010. 2016;31(7):1588–609. doi: 10.1093/humrep/dew082 . 27207175

[6] Xiang C, Zixue S, Shuaipeng Z, Xinghua Z, Jianshe C, Rui W, et al. Yishen Tongluo Recipe combined with minimally invasive surgery for the treatment of varicocele-associated asthenospermia. National Journal of Andrology. 2020;26(4):341–5. 33351302

[7] Hopkins AJNcb. Network pharmacology: the next paradigm in drug discovery. 2008;4(11):682–90. doi: 10.1038/nchembio.118 . 18936753

[8] Nueno VJCpd. Editorial: Towards the Integration of Quantitative and Systems Pharmacology into Drug Discovery: a Systems Level Understanding of Therapeutic and Toxic Effects of Drugs. 2016;22(46):6881–4. doi: 10.2174/138161282246170124192058 . 28236677

[9] Li S, Zhang BJCjonm. Traditional Chinese medicine network pharmacology: theory, methodology and application. 2013;11(2):110–20. doi: 10.1016/s1875-5364(13)60037-0 . 23787177

[10] Zhang Z, Yi P, Yang J, Huang J, Xu P, Hu M, et al. Integrated network pharmacology analysis and serum metabolomics to reveal the cognitive improvement effect of Bushen Tiansui formula on Alzheimer’s disease. 2020;249:112371. doi: 10.1016/j.jep.2019.112371 . 31683034

[11] Lu J, Yan J, Yan J, Zhang L, Chen M, Chen Q, et al. Network pharmacology based research into the effect and mechanism of Xijiao Dihuang decoction against sepsis. 2020;122:109777. doi: 10.1016/j.biopha.2019.109777 . 31918261

[12] Ru J, Li P, Wang J, Zhou W, Li B, Huang C, et al. TCMSP: a database of systems pharmacology for drug discovery from herbal medicines. 2014;6:13. doi: 10.1186/1758-2946-6-13 . 24735618 PMC4001360

[13] Liu Z, Guo F, Wang Y, Li C, Zhang X, Li H, et al. BATMAN-TCM: a Bioinformatics Analysis Tool for Molecular mechANism of Traditional Chinese Medicine. 2016;6:21146. doi: 10.1038/srep21146 . 26879404 PMC4754750

[14] Tao W, Xu X, Wang X, Li B, Wang Y, Li Y, et al. Network pharmacology-based prediction of the active ingredients and potential targets of Chinese herbal Radix Curcumae formula for application to cardiovascular disease. 2013;145(1):1–10. doi: 10.1016/j.jep.2012.09.051 . 23142198

[15] Xu X, Zhang W, Huang C, Li Y, Yu H, Wang Y, et al. A novel chemometric method for the prediction of human oral bioavailability. 2012;13(6):6964–82. doi: 10.3390/ijms13066964 . 22837674 PMC3397506

[16] Zhang S, Li X, Yang XJJoe. Drug-likeness prediction of chemical constituents isolated from Chinese materia medica Ciwujia. 2017;198:131–8. doi: 10.1016/j.jep.2017.01.002 . 28065780

[17] Stelzer G, Plaschkes I, Oz-Levi D, Alkelai A, Olender T, Zimmerman S, et al. VarElect: the phenotype-based variation prioritizer of the GeneCards Suite. 2016:444. doi: 10.1186/s12864-016-2722-2 . 27357693 PMC4928145

[18] Piñero J, Bravo À, Queralt-Rosinach N, Gutiérrez-Sacristán A, Deu-Pons J, Centeno E, et al. DisGeNET: a comprehensive platform integrating information on human disease-associated genes and variants. 2017;45:D833–D9. doi: 10.1093/nar/gkw943 . 27924018 PMC5210640

[19] Szklarczyk D, Morris J, Cook H, Kuhn M, Wyder S, Simonovic M, et al. The STRING database in 2017: quality-controlled protein-protein association networks, made broadly accessible. 2017;45:D362–D8. doi: 10.1093/nar/gkw937 . 27924014 PMC5210637

[20] Shannon P, Markiel A, Ozier O, Baliga N, Wang J, Ramage D, et al. Cytoscape: a software environment for integrated models of biomolecular interaction networks. 2003;13(11):2498–504. doi: 10.1101/gr.1239303 . 14597658 PMC403769

[21] Lu H, Yao J, Yang X, Han J, Wang J, Xu K, et al. Identification of a potentially functional circRNA-miRNA-mRNA regulatory network for investigating pathogenesis and providing possible biomarkers of bladder cancer. 2020;20:31. doi: 10.1186/s12935-020-1108-3 . 32015691 PMC6990554

[22] Primmer C, Papakostas S, Leder E, Davis M, Ragan MJMe. Annotated genes and nonannotated genomes: cross-species use of Gene Ontology in ecology and evolution research. 2013;22(12):3216–41. doi: 10.1111/mec.12309 . 23763602

[23] Kanehisa M, Furumichi M, Tanabe M, Sato Y, Morishima KJNar. KEGG: new perspectives on genomes, pathways, diseases and drugs. 2017;45:D353–D61. doi: 10.1093/nar/gkw1092 . 27899662 PMC5210567

[24] Zhou Y, Zhou B, Pache L, Chang M, Khodabakhshi A, Tanaseichuk O, et al. Metascape provides a biologist-oriented resource for the analysis of systems-level datasets. 2019;10(1):1523. doi: 10.1038/s41467-019-09234-6 . 30944313 PMC6447622

[25] Yu G, Wang L, Han Y, He QJOajoib. clusterProfiler: an R package for comparing biological themes among gene clusters. 2012;16(5):284–7. doi: 10.1089/omi.2011.0118 . 22455463 PMC3339379

[26] Xu W, Yang K, Jiang L, Hu J, Zhou XJFip. Integrated Modules Analysis to Explore the Molecular Mechanisms of Phlegm-Stasis Cementation Syndrome with Ischemic Heart Disease. 2018;9:7. doi: 10.3389/fphys.2018.00007 . 29403392 PMC5786858

[27] Zhu Q, Liu M, He Y, Yang BJAc, nanomedicine, biotechnology. Quercetin protect cigarette smoke extracts induced inflammation and apoptosis in RPE cells. 2019;47(1):2010–5. doi: 10.1080/21691401.2019.1608217 . 31122072

[28] Yang Z, Zhang X, Chen Z, Hu CJE-bc, eCAM am. Effect of Wuzi Yanzong on Reproductive Hormones and TGF-β1/Smads Signal Pathway in Rats with Oligoasthenozoospermia. 2019;2019:7628125. doi: 10.1155/2019/7628125 . 31118967 PMC6500641

[29] Sun W, Guan Q, Wen J, Zhang Q, Yang W, Zhang B, et al. Calcium- and integrin-binding protein-1 is down-regulated in the sperm of patients with oligoasthenozoospermia: CIB1 expression in patients with oligoasthenozoospermia. 2014;31(5):541–7. doi: 10.1007/s10815-014-0177-4 . 24464679 PMC4016369

[30] Luo TT, Lu Y, Yan SK, Xiao X, Rong XL, Guo J. Network Pharmacology in Research of Chinese Medicine Formula: Methodology, Application and Prospective. Chin J Integr Med. 2020;26(1):72–80. Epub 2019/04/04. doi: 10.1007/s11655-019-3064-0 . 30941682

[31] MEN Bo, SUN Zixue, Hui Z, Yanli S, Xiang C, Gaoli H, et al. Yishen Tongluo Decoction on Sperm DNA Integrity of Male Infertility w ith Varicocele for 30 Cases. Chinese Medicine Modern Distance Education of China. 2017;15:84–6. doi: 10.3969/.

[32] Wang Y, Quan F, Cao Q, Lin Y, Yue C, Bi R, et al. Quercetin alleviates acute kidney injury by inhibiting ferroptosis. 2021;28:231–43. doi: 10.1016/j.jare.2020.07.007 . 33364059 PMC7753233

[33] Rakha B, Qurrat-Ul-Ain, Ansari M, Akhter S, Akhter A, Awan M, et al. Gallus gallus murghiEffect of Quercetin on Oxidative Stress, Mitochondrial Activity, and Quality of Indian Red Jungle Fowl () Sperm. 2020;18(4):311–20. doi: 10.1089/bio.2020.0007 . 32522018

[34] Diao R, Gan H, Tian F, Cai X, Zhen W, Song X, et al. In vitro antioxidation effect of Quercetin on sperm function from the infertile patients with leukocytospermia. 2019;82(3):e13155. doi: 10.1111/aji.13155 . 31166052

[35] Cemeli E, Schmid T, Anderson DJE, mutagenesis m. Modulation by flavonoids of DNA damage induced by estrogen-like compounds. 2004;44(5):420–6. doi: 10.1002/em.20071 . 15540192

[36] Yahyazadeh A, Altunkaynak BJB, Commission hopotBS. Protective effects of luteolin on rat testis following exposure to 900 MHz electromagnetic field. 2019;94(4):298–307. doi: 10.1080/10520295.2019.1566568 . 30669870

[37] Potnuri A, Allakonda L, Lahkar MJB, Biomedecine p, pharmacotherapie. Crocin attenuates cyclophosphamide induced testicular toxicity by preserving glutathione redox system. 2018;101:174–80. doi: 10.1016/j.biopha.2018.02.068 . 29486335

[38] Ye R, Yang J, Hai D, Liu N, Ma L, Lan X, et al. Interplay between male reproductive system dysfunction and the therapeutic effect of flavonoids. 2020;147:104756. doi: 10.1016/j.fitote.2020.104756 . 33069836

[39] Zhang Y, Song M, Rui X, Pu S, Li Y, Li C. Supplemental dietary phytosterin protects against 4-nitrophenol-induced oxidative stress and apoptosis in rat testes. Toxicol. 2015;2:664–76. doi: 10.1016/j.toxrep.2015.04.007 28962402 PMC5598167

[40] Martínez P, Proverbio F, Camejo MJAjoa. Sperm lipid peroxidation and pro-inflammatory cytokines. 2007;9(1):102–7. doi: 10.1111/j.1745-7262.2007.00238.x . 17187161

[41] Politch J, Tucker L, Bowman F, Anderson DJHr. Concentrations and significance of cytokines and other immunologic factors in semen of healthy fertile men. 2007;22(11):2928–35. doi: 10.1093/humrep/dem281 . 17855405

[42] Fraczek M, Sanocka D, Kamieniczna M, Kurpisz MJJoa. Proinflammatory cytokines as an intermediate factor enhancing lipid sperm membrane peroxidation in in vitro conditions. 2008;29(1):85–92. doi: 10.2164/jandrol.107.003319 . 17804865

[43] Fraczek M, Szumala-Kakol A, Dworacki G, Sanocka D, Kurpisz MJJori. In vitro reconstruction of inflammatory reaction in human semen: effect on sperm DNA fragmentation. 2013;100(1):76–85. doi: 10.1016/j.jri.2013.09.005 . 24344359

[44] Perdichizzi A, Nicoletti F, La Vignera S, Barone N, D’Agata R, Vicari E, et al. Effects of tumour necrosis factor-alpha on human sperm motility and apoptosis. 2007;27(2):152–62. doi: 10.1007/s10875-007-9071-5 . 17308869

[45] Silva EJR, Ribeiro CM, Mirim AFM, Silva AAS, Romano RM, Hallak J, et al. Lipopolysaccharide and lipotheicoic acid differentially modulate epididymal cytokine and chemokine profiles and sperm parameters in experimental acute epididymitis. Sci Rep. 2018;8(1):103. Epub 2018/01/10. doi: 10.1038/s41598-017-17944-4 ; PubMed Central PMCID: PMC5758752. 29311626 PMC5758752

[46] Hu P, Guan K, Feng Y, Ma C, Song H, Li Y, et al. miR-638 Inhibits immature Sertoli cell growth by indirectly inactivating PI3K/AKT pathway via SPAG1 gene. Cell Cycle. 2017;16(23):2290–300. Epub 2017/11/10. doi: 10.1080/15384101.2017.1380130 ; PubMed Central PMCID: PMC5788492. 29119857 PMC5788492

[47] Cooke PS, Nanjappa MK, Ko C, Prins GS, Hess RA. Estrogens in Male Physiology. Physiol Rev. 2017;97(3):995–1043. Epub 2017/05/26. doi: 10.1152/physrev.00018.2016 ; PubMed Central PMCID: PMC6151497. 28539434 PMC6151497

[48] Wang J, Nuite M, McAlindon TJL. Association of estrogen and aromatase gene polymorphisms with systemic lupus erythematosus. 2010;19(6):734–40. doi: 10.1177/0961203309359517 . 20305046 PMC3653634

[49] Saraswat S, Kharche SD, Rout PK, Pawaiya R, Gangwar C, Swain DK, et al. Molecular expression and identification of caprine estrogen receptor gene 1 for fertility status in bucks. Reprod Domest Anim. 2020;55(9):1080–92. Epub 2020/06/13. doi: 10.1111/rda.13746 . 32531861

[50] Nordkap L, Almstrup K, Nielsen JE, Bang AK, Priskorn L, Krause M, et al. Possible involvement of the glucocorticoid receptor (NR3C1) and selected NR3C1 gene variants in regulation of human testicular function. Andrology. 2017;5(6):1105–14. doi: 10.1111/andr.12418 28992366

[51] Shen X, Niu C, Guo J, Xia M, Xia J, Hu Y, et al. Stra8 may inhibit apoptosis during mouse spermatogenesis via the AKT signaling pathway. International Journal of Molecular Medicine. 2018. doi: 10.3892/ijmm.2018.3825 30106128

[52] Deng C, Lv M, Luo B, Zhao S, Mo Z, Xie YJCmm. The role of the PI3 K/AKT/mTOR signalling pathway in male reproduction. 2020. doi: 10.2174/1566524020666201203164910 . 33272176

[53] Rashid H, Yadav R, Kim H, Chae HJA. ER stress: Autophagy induction, inhibition and selection. 2015;11(11):1956–77. doi: 10.1080/15548627.2015.1091141 . 26389781 PMC4824587

[54] Zhang J, Yao Y, Pan J, Guo X, Han X, Zhou J, et al. Maternal exposure to Di-(2-ethylhexyl) phthalate (DEHP) activates the PI3K/Akt/mTOR signaling pathway in F1 and F2 generation adult mouse testis. Exp Cell Res. 2020;394(2):112151. Epub 2020/06/27. doi: 10.1016/j.yexcr.2020.112151 . 32589889

[55] Huang W, Cao Z, Zhang J, Ji Q, Li Y. Aflatoxin B1 promotes autophagy associated with oxidative stress-related PI3K/AKT/mTOR signaling pathway in mice testis. Environ Pollut. 2019;255(Pt 2):113317. Epub 2019/10/15. doi: 10.1016/j.envpol.2019.113317 . 31610502

[56] Lu T, Ling C, Hu M, Meng X, Deng Y, An H, et al. Effect of Nano-Titanium Dioxide on Blood-Testis Barrier and MAPK Signaling Pathway in Male Mice. 2020. doi: 10.1007/s12011-020-02404-4 . 32990870

[57] Wang H, Zhou W, Zhang J, Li HJBr. Role of JNK and ERK1/2 MAPK signaling pathway in testicular injury of rats induced by di-N-butyl-phthalate (DBP). 2019;52(1):41. doi: 10.1186/s40659-019-0248-1 . 31387634 PMC6685163

[58] Yu L, Yang X, Ma B, Ying H, Shang X, He B, et al. Abnormal arachidonic acid metabolic network may reduce sperm motility via P38 MAPK. 2019;9(4):180091. doi: 10.1098/rsob.180091 . 31014201 PMC6501647

[59] Bai X, Tang Y, Li Q, Chen Y, Liu D, Liu G, et al. Network pharmacology integrated molecular docking reveals the bioactive components and potential targets of Morinda officinalis-Lycium barbarum coupled-herbs against oligoasthenozoospermia. Sci Rep. 2021;11(1):2220. Epub 2021/01/28. doi: 10.1038/s41598-020-80780-6 ; PubMed Central PMCID: PMC7838196. 33500463 PMC7838196

