# Supplementary material for: A network pharmacology approach to determine the underlying mechanisms of action of Yishen Tongluo formula for the treatment of oligoasthenozoospermia
Source: PLoS One. 2021 Jun 21;16(6):e0252906. doi: 10.1371/journal.pone.0252906 (PMC8216565; doi:10.1371/journal.pone.0252906)
Supplement: Attachment — Submitted filename: Response to Reviewers.docx [file pone.0252906.s001.docx]

Dear Editor：

Thank you for your letter and for the reviewers’ comments concerning our manuscript entitled “A Network Pharmacology Approach to the Underlying Mechanisms of Action of Yishen Tongluo Formula for the Treatment of Oligoasthenozoospermia” (ID: PONE-D-21-02516). Those comments are all valuable and very helpful for revising and improving our paper, as well as the important guiding significance to our manuscript. We have studied comments carefully and have made corrections which we hope meet with approval. In this revised version, changes to our manuscript within the document were all highlighted by using red colored text. Point-to-point responses to the reviewers are listed below this letter.

**Additional Editor Comments:**
Dear Dr. Zixue Sun (Author),
After careful reviewing of this manuscripts by keen reviewers from the field, I think this manuscript can be published after addressing comments by reviewers. In specific, the manuscript need improvement in language and writing, preferably by a native English speaker. In addition, representing data need corrections and improvements as mentioned in comments.
**Reply**: This document certifies that the manuscript “A Network Pharmacology Approach to the Underlying Mechanisms of Action of Yishen Tongluo Formula for the Treatment of Oligoasthenozoospermia” prepared by the authors Yangdi Chen, Fanggang Bi, Zixue Sun was edited for proper English language, grammar, punctuation, spelling, and overall style by one or more of the highly qualified native English-speaking editors at SNAS. This certificate was issued on May 13, 2021 and may be verified on the SNAS website using the verification code F84C-D686-DCDD-B7C0-AF45.

**Responds to the reviewer’s comments:**

**Reviewer #1:** This study is solely based on ‘putative’ targets, ‘potential’ targets/mechanisms retrieved from databases and analyzed to make connections. Spermatogenesis is one of the fundamental process that sustains life and, undoubtedly, there will be hundreds of molecular mechanisms regulating such processes. Mere bioinformatics analysis of ‘putative’ targets and linking them to ‘potential’ mechanisms does not warrant publication of this paper in PlosOne. Needless to comment on grammatical and typographical errors throughout the text.

**Reply**: Thank you for your comments. This study, which is based on ‘putative’ targets, ‘potential’ targets/mechanisms retrieved from databases and analyzed to make connections, has a limitation, and we also point it out at the end of the article. However, network pharmacological methods can identify the probable most effective compounds and bioactive ingredients of YSTLF in treating oligoasthenozoospermia, their possible mode of actions and suggested that network pharmacology prediction may be a helpful tool to illustrate the interactive relationship between multi-components, multi-targets and multi-pathways of bioactive compounds in traditional Chinese herbal medicine towards evaluating their compatibility and rationality. Xue Bai et al.’s research(Bai X, Tang Y, Li Q, Chen Y, Liu D, Liu G, et al. Network pharmacology integrated molecular docking reveals the bioactive components and potential targets of Morinda officinalis-Lycium barbarum coupled-herbs against oligoasthenozoospermia. Sci Rep. 2021;11(1):2220. Epub 2021/01/28. doi: 10.1038/s41598-020-80780-6. PubMed PMID: 33500463; PubMed Central PMCID: PMCPMC7838196.), which was published in the journal Scientific Reports, demonstrated that *Morinda offcinalis–Lycium barbarum* coupled-herbs (MOLBCH) alleviated apoptosis, promoted male reproductive function, and reduced oxidant stress in the treatment of OA. Ohioensin-A, quercetin, beta-sitosterol and sitosterol were the key bioactive components. Androgen receptor (AR), Estrogen receptor (ESR1), Mitogen-activated protein kinase 3 (MAPK3), RAC-alpha serine/threonine-protein kinase (AKT1), Glyceraldehyde-3-phosphate dehydrogenase (GAPDH) were the core potential targets. PI3K/Akt signaling pathway, prostate cancer, AGE-RAGE signaling pathway in diabetic complications were the most representative pathways. Their research methods are almost the same as ours. So we think that our research has merit. These observations provide a reliable basis for future experiments and deeper insight into the pathogenesis of oligoasthenozoospermia, and can develop new therapeutic instructions to treat oligoasthenozoospermia.

**Reviewer #2:**

**Comment 1**. Figures 4 & 6 - in the PDF version I have received from the journal - are NOT clear. I could hardly read the labels. This bad resolution is NOT suitable for publications. In my opinion, the original figures have to be enhanced to be not less than 900 dpi, so that they could be readable in PDF version.

**Reply and modifications**: Thank you for your valuable comments. However, PLOS ONE's figure file requirements are 300 – 600 dpi in resolution, <10 MB in file size. We have endeavoured to revise Figures 4 & 6 to meet the publication requirements and uploaded new Figures 4 & 6.

**Comment 2**. It is obvious for any reader that the words “we” & “our” were repeated in many sentences. I think it could be more convenient to rephrase such sentences to be in passive voice, which is more suitable for scientific writing.

**Reply and modifications**: In the revised version, we have changed the active voice to the passive voice to be more suitable for scientific writing.

**Reviewer #3:** Thank you for the opportunity to review the manuscript with the objective to utilize network pharmacology approach to elucidate chemical and pharmacological profiles of Yishen Tongluo formula (YSTLF) composed of seven herbs of traditional Chinese medicine origin and to explore the potential mechanisms of action of YSTLF on oligoasthenozoospermia.
The study is based on the theories of multi-directional pharmacology and systems biology, and how network pharmacology could be used to construct complex network models to study the biological or pharmacological properties of the multi-component formula and explore its physiological or pharmacological mechanisms. It was supported by elaborate data analysis, virtual computing modelling technology and network public databases. It identified the probable most effective compounds and bioactive ingredients of YSTLF in treating oligoasthenozoospermia, their possible mode of actions and suggested that network pharmacology prediction may be a helpful tool to illustrate the interactive relationship between multi-components, multi-targets and multi-pathways of bioactive compounds in traditional Chinese herbal medicine towards evaluating their compatibility and rationality.
The methods used were sufficiently described for another researcher to reproduce the study with the same or similar methods.
The study limitations and areas of further research were highlighted.

**Reply:** Thank you very much for your comments. Thank you for your affirmation and support for our research.

**Reviewer #4:**

**Comment 1**. Language editing.

**Reply**: This document certifies that the manuscript “A Network Pharmacology Approach to the Underlying Mechanisms of Action of Yishen Tongluo Formula for the Treatment of Oligoasthenozoospermia” prepared by the authors Yangdi Chen, Fanggang Bi, Zixue Sun was edited for proper English language, grammar, punctuation, spelling, and overall style by one or more of the highly qualified native English-speaking editors at SNAS. This certificate was issued on May 13, 2021 and may be verified on the SNAS website using the verification code F84C-D686-DCDD-B7C0-AF45.

**Comment 2**. Be more specific with the results obtained in the clinical trial using YSTLF in conjunction with the surgical procedure in the treatment of oligoasthenozoospermia.

**Reply and modifications**: We have added the data of the above research result in the paper (Page 5, line 99) and cited literatures in the discussion section (Page 15, lines 316-319) in the file labeled 'Revised Manuscript with Track Changes'.

**Comment 3**. Generate a discussion that could be enriched with the results presented by the recent publication with the title: Network pharmacology integrated molecular docking reveals the bioactive components and potential targets of *Morinda officinalis – Lycium barbarum* coupled-herbs against oligoasthenozoospermia.
**Reply and modifications**: Thank you for your suggestion. We have cited this literature in the discussion section (Page 20, lines 425-432) to make our study more persuasive.

**Comment 4**. Possibly the development of a diagram that represents the possible mechanisms involved.

**Reply and modifications**: Thank you for your suggestion. We have added diagrams, which show the targets related to these three most important signaling pathways (PI3K/Akt, MAPK and Apoptosis signaling pathways) during the process of YSTLF in treating oligoasthenozoospermia. The above can represent the possible mechanisms involved.

If there are any other modifications we could make, we would like very much to modify them and we really appreciate your help. Thank you very much for your help.

Best wishes!

Zixue Sun

Department of Reproductive Medicine, Henan Province Hospital of Traditional Chinese Medicine (The Second Affiliated Hospital of Henan University of Chinese Medicine)

No.6 Dongfeng Road, Jinshui District, Zhengzhou, China, 450002
